# Characterization of the complete genome sequence of the recombinant norovirus GII.P16/GII.4_Sydney_2012 revealed in Russia

**DOI:** 10.18699/VJ20.597

**Published:** 2020-02

**Authors:** E.V. Zhirakovskaia, A.Y. Tikunov, S.N. Sokolov, B.I. Kravchuk, E.I. Krasnova, N.V. Tikunova

**Affiliations:** Institute of Сhemical Biology аnd Fundamental Medicine of Siberian Branch of the Russian Academy of Sciences, Novosibirsk, Russia; Institute of Сhemical Biology аnd Fundamental Medicine of Siberian Branch of the Russian Academy of Sciences, Novosibirsk, Russia; Institute of Сhemical Biology аnd Fundamental Medicine of Siberian Branch of the Russian Academy of Sciences, Novosibirsk, Russia State Research Center of Virology and Biotechnology Vector, Koltsovo, Novosibirsk region, Russia; Institute of Сhemical Biology аnd Fundamental Medicine of Siberian Branch of the Russian Academy of Sciences, Novosibirsk, Russia; Novosibirsk State Medical University, Department of Infectious Diseases, Novosibirsk, Russia; Institute of Сhemical Biology аnd Fundamental Medicine of Siberian Branch of the Russian Academy of Sciences, Novosibirsk, Russia

**Keywords:** norovirus, complete genome, polymerase, protein p48, capsid proteins, phylogenetic analysis, acute gastroenteritis, monitoring of genotypes, норовирус, полный геном, полимераза, белок p48, капсидные белки, филогенетический анализ, острый гастроэнтерит, мониторинг генотипов

## Abstract

Noroviruses (the Caliciviridae family) are a common cause of acute gastroenteritis in all age groups. These small non-envelope viruses with a single-stranded (+)RNA genome are characterized by high genetic variability. Continuous changes in the genetic diversity of co-circulating noroviruses and the emergence of new recombinant variants are observed worldwide. Recently, new recombinant noroviruses with a novel GII.P16 polymerase associated with different capsid proteins VP1 were reported. As a part of the surveillance study of sporadic cases of acute gastroenteritis in Novosibirsk, a total of 46 clinical samples from children with diarrhea were screened in 2016. Norovirus was detected in six samples from hospitalized children by RT-PCR. The identified noroviruses were classified as recombinant variants GII.P21/GII.3, GII. Pe/GII.4_Sydney_2012, and GII.P16/GII.4_Sydney_2012 by sequencing of the ORF1/ORF2 junction. In Novosibirsk, the first appearance of the new recombinant genotype GII.P16/ GII.4_Sydney_2012 was recorded in spring 2016. Before this study, only four complete genome sequences of the Russian GII.P16/GII.3 norovirus strains were available in the GenBank database. In this work, the complete genome sequence of the Russian strain Hu/GII.P16-GII.4/RUS/Novosibirsk/NS16-C38/2016 (GenBank KY210980) was determined. A comparison of the nucleotide and the deduced amino acid sequences showed a high homology of the Russian strain with GII.P16/GII.4_Sydney_2012 strains from other parts of the world. A comparative analysis showed that several unique substitutions occurred in the GII.P16 polymerase, N-terminal p48 protein, and minor capsid protein VP2 genes, while no unique changes in the capsid VP1 gene were observed. A functional significance of these changes suggests that a wide distribution of the strains with the novel GII.P16 polymerase may be associated both with several amino acid substitutions in the polymerase active center and with the insertion of glutamic acid or glycine in an N-terminal p48 protein that blocks the secretory immunity of intestinal epithelial cells. Further monitoring of genotypes will allow determining the distribution of norovirus recombinants with the polymerase GII.P16 in Russia.

## Abstract (ru)

Норовирусы (сем. Caliciviridae) считаются частой причиной острого гастроэнтерита у людей всех возрастов. Эти небольшие безоболочечные вирусы с одноцепочечным (+)РНК-геномом характеризуются высокой генетической вариабельностью. По всему миру наблюдается постоянное изменение генетического разнообразия циркулирующих норовирусов и появление новых рекомбинантных вариантов. Недавно опубликованы данные о распространении рекомбинантных штаммов норовируса, в которых новая полимераза генотипа GII.P16 сочеталась с капсидными белками VP1 разных генотипов. В рамках мониторинга спорадических случаев острых гастроэнтеритов в Новосибирске в 2016 г. было протестировано 46 клинических образцов от детей с диареей. Методом ОТ-ПЦР норовирус детектирован в шести клинических образ- цах от госпитализированных детей. Выявленные норовирусы путем секвенирования региона перекрывания ORF1/ORF2 были классифицированы как рекомбинантные варианты GII.P21/GII.3, GII.Pe/GII.4_Sydney_2012 и GII. P16/ GII.4_Sydney_2012. Появление нового рекомбинантного генотипа GII. P16/GII.4_Sydney_2012 впервые зафиксировано в Новосибирске весной 2016 г. До этого исследования в базе данных GenBank было доступно всего четыре полногеномные последовательности российских штаммов норовируса генотипа GII.P16/ GII.3. В настоящей работе была определена полная последовательность генома российского штамма Hu/GII.P16- GII.4/RUS/Novosibirsk/NS16-C38/2016 (GenBank KY210980). Сравнение нуклеотидной и выведенной амино- кислотной последовательностей показало высокую гомологию этого российского штамма со штаммами ге- нотипа GII.P16/GII.4_Sydney_2012 из других регионов мира. Сравнительный анализ показал, что уникальные замены произошли в последовательностях генов полимеразы генотипа GII.P16, N-терминального белка p48 и минорного капсидного белка VP2, при этом существенных изменений в гене основного капсидного белка VP1 не наблюдалось. Анализ функциональной значимости этих изменений позволил предположить, что ши- рокое распространение штаммов с новой полимеразой GII.P16, возможно, связано как с несколькими ами- нокислотными заменами в активном центре полимеразы, так и со вставкой остатка глутаминовой кислоты или глицина в N-терминальном белке p48, который блокирует секреторный иммунитет эпителиальных кле- ток кишечника. Дальнейший мониторинг генотипов позволит оценить распространение рекомбинантных норовирусов с полимеразой GII.P16 на территории России.

## Introduction

Noroviruses (Caliciviridae family, Norovirus genus) are considered to be one of the common causes of outbreaks and sporadic cases of acute gastroenteritis (AGE) in humans of all ages (Bartsch et al., 2016). Norovirus infection can cause severe outcomes of the disease in very young and elderly individuals, as well as chronic diarrhea, lasting from several months to several years, in immunocompromised and cancer patients, and humans after organ transplantation (Brown et al., 2017; Woodward et al., 2017; Petrignani et al., 2018). Due to the low infectious dose (~10–100 viral particles) and high resistance in the environment, noroviruses are rapidly transmitted person-to-person, by food and water (Kirby et al., 2015; Towers et al., 2018). A meta-analysis of epidemiological data from many countries showed that the incidence of norovirus infection among patients with AGE regardless of their age was 17–20 % in 2008–2014 (Ahmed et al., 2014). The prevalence of asymptomatic norovirus infection is estimated from 4 to 18 % in different regions (Qi et al., 2018).

The polyadenylated single-stranded (+)RNA genome of norovirus (~7.5 kb) contains three overlapping open reading frames (ORF1–ORF3) (Green, 2013). ORF1 encodes a large polyprotein that is post-translationally cleaved by viral protease into six nonstructural proteins, including RNAdependent RNA polymerase (RdRp); ORF2 and ORF3 encode major (VP1) and minor (VP2) capsid proteins, respectively. Two mechanisms of norovirus genetic variability have been identified: point mutations and recombination (Bull, White, 2011). Due to recombination events occurring in the norovirus genome near the overlapping region of the 3ʹ-end of ORF1 (RdRp) and 5ʹ-end of ORF2 (VP1), a dual nomenclature of noroviruses defining the RdRp/VP1 genotypes was recently developed (Kroneman et al., 2013).

Noroviruses exhibit significant genetic and antigenic diversity. Based on the VP1 amino acid sequence, noroviruses are currently classified into at least seven genogroups (GI–GVII), which are further sub-divided into more than forty genotypes (Kroneman et al., 2013). It has been established that GI, GII and GIV noroviruses can cause disease in humans (Green, 2013; Parra et al., 2017). In the most common genogroup GII, at least 31 RdRp genotypes and 23 VP1 genotypes are distinguished, and their combination is designated as GII.Px/GII.x (Kroneman et al., 2013; Vinje, 2015; RIVM, https://www.rivm.nl/mpf/typingtool/norovirus/). The average duration of genotype-specific immunity after norovirus infection can be from 4 to 8 years (Simmons et al., 2013), however, due to the existence of a wide range of genetic variants, subsequent norovirus infection with other antigenic variants or “immunotypes” can occur in a shorter time (Parra et al., 2017).

Since the 1990s, norovirus GII.4 was considered to be predominant and several epidemic variants of GII.P4/GII.4 replaced each other at intervals of 2–3 years for two decades (Eden et al., 2013; Hoa Tran et al., 2013). In 2012, a new recombinant GII.4 norovirus classified as GII.Pe/GII.4_ Sydney_2012 appeared and later became the dominant strain worldwide (van Beek et al., 2013). However, changes in the molecular epidemiology of norovirus have been observed in recent years. In the winter season 2014/2015, a new GII.P17/ GII.17 strain, which was first registered in China, quickly replaced the GII.Pe/GII.4_Sydney_2012 variant and initially spread to Asia, and later, to other regions (de Graaf et al., 2015). Recently, the prevalence of new recombinant norovirus strains with the GII.P16 polymerase associated with multiple VP1 genotypes, including GII.4_Sydney_2012, has been reported in different regions (Barreira et al., 2017; Bidalot et al., 2017; Ruiz et al., 2017; Han et al., 2018; Hata et al., 2018).

In Novosibirsk, long-term monitoring of the genetic diversity of enteric viruses showed that noroviruses GII.P4/GII.4 were a common cause of sporadic AGE cases in 2003–2012, while noroviruses with the GII.P16 polymerase were rarely detected (Zhirakovskaia et al., 2015, 2019). In the spring of 2016, we recorded the emergence of a new recombinant variant GII.P16/GII.4_Sydney_2012 in Novosibirsk. Before this study, only four complete genome sequences of recombinant GII.P16/GII.3 noroviruses from Russia were available in the GenBank database (Zhirakovskaia et al., 2015, 2019). The aim of this study was complete genome sequencing of the new Russian GII.P16/GII.4_Sydney_2012 strain and comparative analysis with similar strains from other regions and with Russian 2005–2012 strains in which the GII.P16 polymerase was in association with various other VP1 genotypes.

## Materials and methods

**Origin of virus strains.** As a part of the surveillance study genetic diversity of enteric viruses, clinical samples were collected from children with diarrhea who were hospitalized at Children’s City Clinical Hospital No. 3 and were on outpatient treatment in 2016. Written informed consent was obtained from each parent/guardian of the child to participate in the study, in compliance with voluntariness in accordance with the Federal Law “On the Principles of the Protection of Citizens’ Health in the Russian Federation”. Detection and differentiation of viral RNA were performed by RT-PCR using a verified laboratory primer panel, as previously described (Zhirakovskaia et al., 2019).

**Sequencing.** The detected noroviruses were characterized by sequencing of the genome region (~1400 nt), including the ORF1/ORF2 junction (20 nt). The nucleotide sequences were determined by the Sanger method using the BigDye™ Terminator v.3.1 Cycle Sequencing Kit and 3500 Genetic Analyzer (Applied Biosystems, CA, USA). The complete genome sequencing of the strain Hu/GII.P16-GII.4/RUS/ Novosibirsk/NS16-C38/2016 was performed by the “primerwalking” method using a panel of newly designed primers. The obtained data were analyzed by FinchTV (Geospiza, WA, USA). Partial fragments were assembled into a full-length genome sequence using SeqMan from the Lasergene Evolution Suite software package (DNASTAR, Madison, WI, USA). Norovirus genotype was determined using the Norovirus Typing Tool v. 2.0 (RIVM; https://www.rivm.nl/mpf/typingtool/norovirus/) (Kroneman et al., 2013).

**Phylogenetic analysis.** Reference norovirus sequences were obtained by search on BLAST 2.9.0+ (https://www.ncbi.nlm.nih.gov/). ClustalW alignment and phylogenetic analysis of nucleotide sequences were performed using MEGA 7 (https://www.megasoftware.net/). Phylogenetic trees were constructed using the Neighbor-Joining method with the Kimura 2-parameter model. The analysis was performed with a bootstrap of 1000 replicas; only values >80 % were indicated. The identity of the nucleotide and amino acid sequences was calculated using BioEdit v7.2.6 software (http://www.mbio.ncsu.edu/bioedit/bioedit.html). The variability of the deduced amino acid sequences of the polyprotein comprising GII.P16 RdRp, as well as the capsid proteins VP1 and VP2 of variant GII.4_Sydney_2012 was determined using the Sequence Data Explorer from MEGA 7.

The nucleotide sequences obtained in this study were annotated and deposited in the GenBank database with access numbers KY210919, KY210976–KY210980, KY210983, MG892912 и MG892914.

## Results

A total of 46 fecal samples from children aged 1 month to 8 years were tested by RT-PCR. Enteric viruses were detected in 15 (32.6 %) samples. Norovirus infection was identified in six hospitalized children aged 1 to 9 months. Analysis of nucleotide sequences (~1400 nt), including the ORF1/ORF2 junction, by BLAST (https://www.ncbi.nlm.nih.gov/) and RIVM (https://www.rivm.nl/mpf/typingtool/ norovirus/) showed that the identified noroviruses belonged to three recombinant variants (Table 1).

**Table 1.**
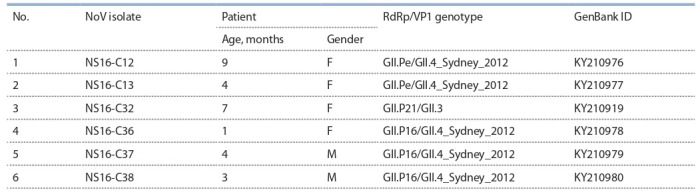
Epidemiological data of norovirus-positive cases of acute gastroenteritis in Novosibirsk, Russia in 2016

The nucleotide sequences of the norovirus strains that were most homologous to the sequences obtained in this study were determined by search on BLAST. Isolate NS16-C32 related to GII.P21/GII.3 genotype had high homology with strains circulating in Novosibirsk (97.6–98.9 %) in 2010–2012 (Zhirakovskaia et al., 2019) and in Europe (96.5–97.2 %) in 2014–2016 (Brown et al., 2019). Two isolates NS16-C12 and NS16-C13 genotyped as GII.Pe/GII.4_Sydney_2012 had a high similarity (99.3–99.5 %) with GII.Pe/GII.4 strains (2014–2016) from Southeast Asia and Great Britain (Brown et al., 2019) and 96.9–97.1 % identity with strains that previously circulated in Novosibirsk (Zhirakovskaia et al., 2019). Nucleotide sequences of three remaining isolates NS16-C36, NS16-C37, and NS16-C38 related to the new genotype GII. P16/GII.4_Sydney_2012 had 100 % identity to each other and 96.2 % homology with isolates NS16-C12 and NS16- C13. Genetic similarity of isolates NS16-C36, NS16-C37 and NS16-C38 with strains GII.P16/GII.4_Sydney_2012 from other regions was 97.4–98.9 %.

Based on complete norovirus sequences available in the GenBank database, several sets of original primers were designed for complete sequencing of ORF1, RdRp, ORF2, and ORF3 of different genotypes. For two isolates, NS16-C13 and NS16-C32, nucleotide sequences (~4300 nt), including RdRp, ORF2 and ORF3, were identified and deposited in the GenBank database as strains Hu/GII.Pe-GII.4/RUS/ Novosibirsk/NS16-C13/2016 (GenBank KY210977) and Hu/GII.P21-GII.3/RUS/Novosibirsk/NS16-C23/2016 (Gen- Bank KY210919).

For isolate NS16-C38, complete genome sequence (7560 nt) including the 3ʹ-untranslated region (47 nt) was determined and deposited in the GenBank database as the strain Hu/GII.P16-GII.4/RUS/Novosibirsk/NS16-C38/2016 (GenBank KY210980). Analysis of the deduced amino acid sequences showed that ORF1 (5100 nt) encoded a polyprotein of 1700 amino acid (aa) residues of length; ORF2 (1623 nt) and ORF3 (807 nt) encoded the capsid proteins VP1 (541 aa) and VP2 (269 aa), respectively. The complete nucleotide sequence of the Russian strain Hu/GII.P16-GII.4/RUS/Novosibirsk/ NS16-C38/2016 had 98–99 % homology with recombinant GII.P16/GII.4_Sydney_2012 strains that appeared in the USA and the UK in the winter season 2015/2016. Since the strain studied was recombinant, a comparative analysis was performed separately for each ORF.

**Comparative analysis of the ORF1**

**Fig. 1.**
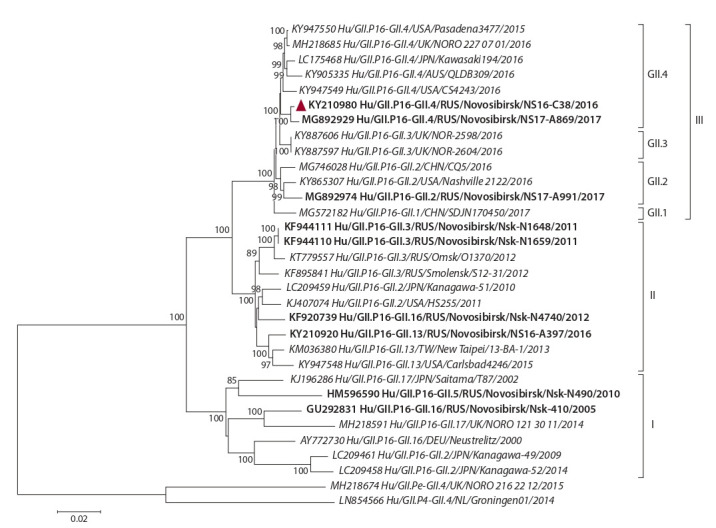
Phylogenetic tree of full (5100 nt) ORF1 sequences of noroviruses with GII.P16 RdRp. Novosibirsk strains are in bold, the analyzed strain is marked with a triangle. Reference strains are indicated in italics; external sequences are strains with polymerase GII.P4 and GII.Pe.

Phylogenetic analysis of complete ORF1 nucleotide sequences with GII.P16 RdRp available in GenBank showed that the analyzed strains were divided into three clusters (I, II and III); separate clades (supported on >85 %) within them were formed by strains with the same VP1 genotype (Fig. 1). Cluster III contained contemporary recombinant strains with GII.P16 RdRp associated with VP1 of four genotypes, GII.1, GII.2, GII.3, and GII.4_Sydney_2012. The ORF1 homology of the Russian strain Hu/GII.P16-GII.4/RUS/Novosibirsk/NS16- C38/2016 with other strains of cluster III was 97.9–99 %.

Comparative analysis showed that the complete ORF1 sequences of the GII.P16 RdRp strains varied from 5094 nt (reference strain AY772730_Hu/GII.P16-GII.16/DEU/ Neustrelitz/2000) to 5100 nt (cluster III). When aligned, the GAA insert in the region encoding the N-terminal protein p48 was found in recombinant GII.P16/GII.17 strains (cluster I) and in all strains from cluster II. Additional insertion of GAA or GGA was detected in contemporary recombinant strains from cluster III in the same region of the p48 gene. For ORF1, 1317 (25.8 %) variable sites were determined, of which 906 (17.7 %) were informative, i. e. were in two or more strains.

For polyprotein, 182 (10.7 %) variable sites were found, 104 (6.1 %) of which were informative (Table 2). Comparative analysis showed that 14 variable sites were unique to the novel GII.P16 RdRp lineage (cluster III), and the substitutions in seven positions 52, 53, 644, 845, 853, 1546, and 1549 resulted in a change in amino acid chemistry. An assessment of the functional significance of the changes revealed that three non-synonymous substitutions (at 52, 53 and 165) and 77E/G insert were found in p48, which plays a role in virus entry through the host cell membrane (Fernandez-Vega et al., 2004). Four of the five non-synonymous substitutions (at 1482, 1521, 1546, and 1549) within GII.P16 RdRp occurred in the active center (see Table 2), and this may have affected the norovirus transmission.

**Table 2.**
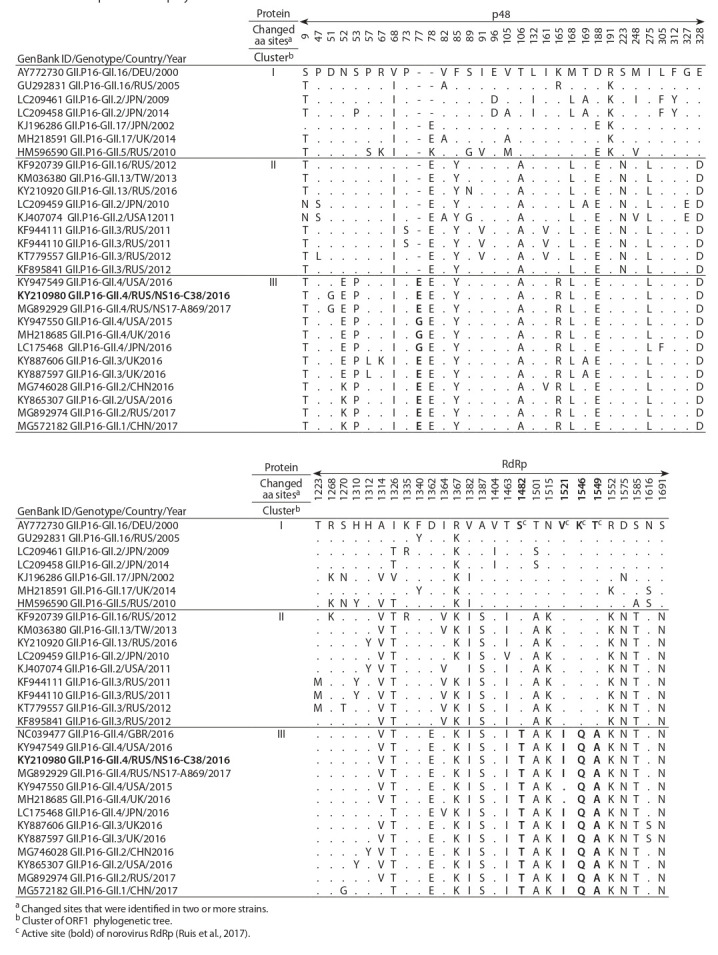
Comparison of deduced amino acid sequences of the N-terminal protein p48 and the RNA-depended RNA polymerase GII.P16 of different norovirus strains

**Comparative analysis of the ORF2**

**Fig. 2.**
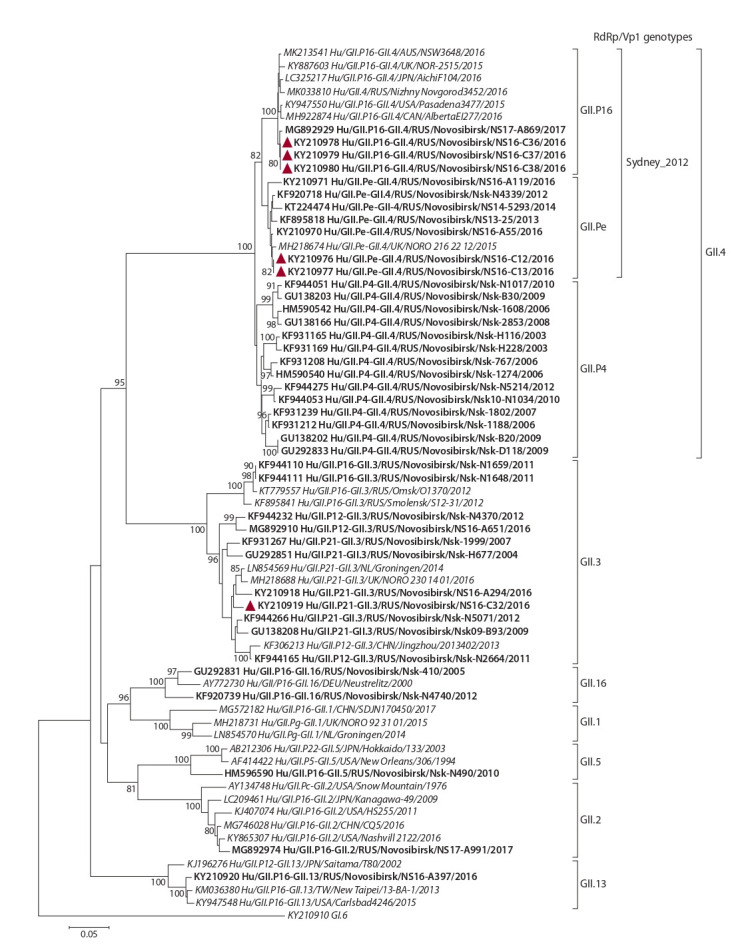
Phylogenetic tree of the partial (~600 nt) ORF2 sequences of GII noroviruses. Novosibirsk strains are in bold, strains 2016 are marked with a triangle. Genotypes of VP1 are noted by brackets to the right. Reference strains are indicated in italics; GI.6 norovirus is an external sequence.

In addition to the sequences determined in this study, phylogenetic analysis of ORF2 included strains of different capsid genotypes, which were identified in combination with the GII. P16 RdRp. On phylogenetic tree of the partial ORF2 sequences, the analyzed strains formed separate clusters in accordance with the capsid genotype and were further subdivided into separate clades depending on the RdRp genotype (Fig. 2).

The GII.4 nucleotide sequences divided into two major clades. The most polymorphic clade includes strains with the GII.P4 RdRp, which previously circulated in Novosibirsk and belonged to six GII.4 epidemic variants: Farmington_ Hills_2002, Hunter_2004, Yerseke_2006a, Den_Haag_2006b, Apeldoorn_2007 and New-Orleans_2009 (Zhirakovskaia et al., 2015). The second clade was formed by GII.4_Sydney_ 2012 strains, which were further divided into two separate clusters, depending on the RdRp genotype – GII.Pe or GII. P16 (supported on 99–100 %).

The ORF2 similarity of the Russian strain Hu/GII.P16- GII.4/RUS/Novosibirsk/NS16-C38/2016 to the GII.P16/GII.4_ Sydney_2012 strains from other regions was 98.8–99 %. Comparison of complete ORF2 sequences of the GII. P16/ GII.4_Sydney_2012 strains with the GII.Pe/GII.4_Sydney_ 2012 and GII.P4/GII.4_New_Orleans_2009 strains revealed 236 (14.5 %) variable sites, of which 132 (8.1 %) were informative. In deduced amino acid sequences of VP1, eight variable sites (at 15, 310, 341, 359, 368, 373, 377, and 396) were unique to the GII.4_Sydney_2012 variant, which distinguished them from the GII.4_New_Orleans_2009 variant, and only two of these changes (at 368 and 373) were located in the hypervariable epitope A (Table 3). Notably, only one variable site at position 540 was unique to the new lineage of GII.P16/GII.4_Sydney_2012, however, it was not located in antigenic regions of the major capsid protein VP1.

**Table 3.**
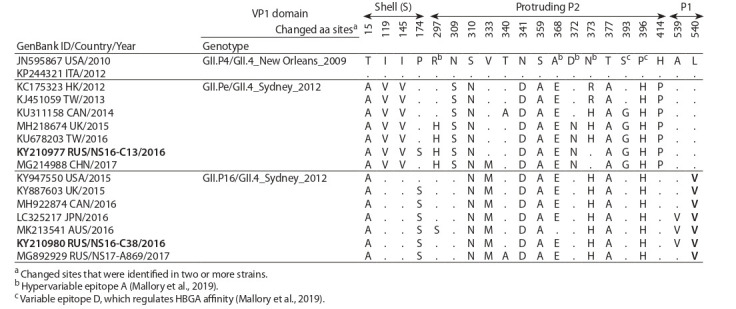
Comparison of deduced VP1 amino acid sequences of norovirus genotypes GII.4_Sydney_2012 and GII.4_New_Orleans_2009

**Comparative analysis of the ORF3**

**Fig. 3.**
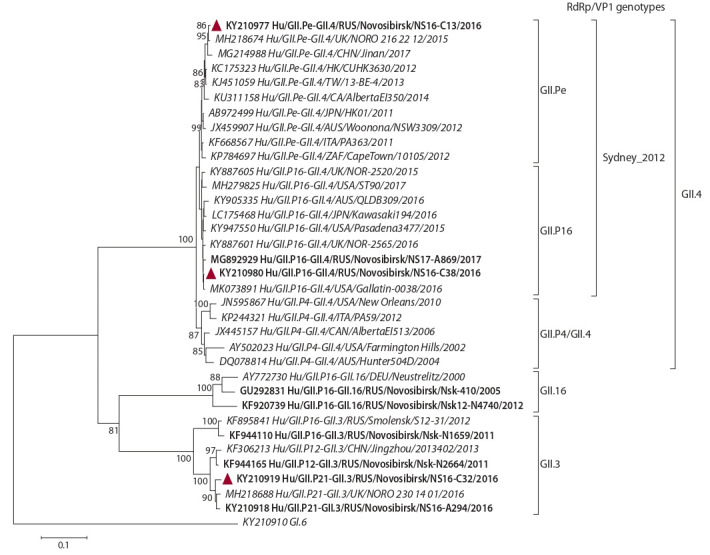
The phylogenetic tree of complete (807 nt) ORF3 sequences of GII noroviruses. Novosibirsk strains are in bold, strains 2016 are marked with a triangle. The RdRp/VP1 genotypes are noted by brackets to the right. Reference strains are indicated in italics; GI.6 norovirus is an external sequence.

Phylogenetic analysis of complete ORF3 sequences of strains with the GII.P16 RdRp showed that the analyzed sequences were divided into separate clusters depending on the VP1 genotype (Fig. 3). Within each VP1 genotype, strains with different RdRp genotypes were grouped into separate clades. Comparative analysis of complete ORF3 sequences of GII.P16/GII.4_Sydney_2012 strains with those of GII.Pe/GII.4_Sydney_2012 and GII.P4/GII.4_New_Orleans_2009 strains revealed 109 (13.5 %) variable sites, and 55 (6.8 %) of them were informative.

In the deduced amino acid sequences of the minor capsid protein VP2 of GII.4_Sydney_2012 strains, eight unique variable sites at positions 81, 108, 148, 149, 158, 164, 205, and 241 were identified (Table 4), which differed them from GII.4_New_Orleans_2009 strains. In addition, two other variable sites (at 155 and 157) were unique to the new lineage of GII.P16/GII.4_Sydney_2012.

**Table 4.**
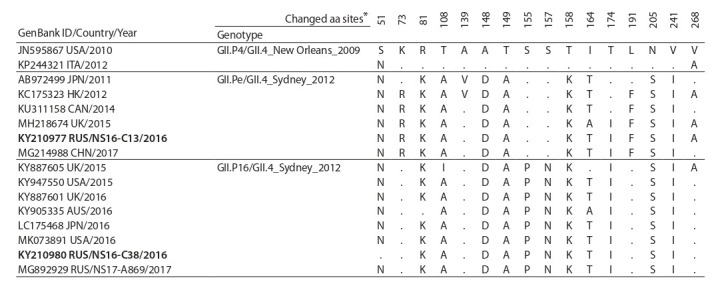
Comparison of deduced VP2 amino acid sequences of norovirus GII.4_Sydney_2012 and GII.4_New_Orleans_2009

## Discussion

This work is a part of long-term monitoring of the genetic diversity of noroviruses associated with sporadic AGE cases in Novosibirsk, Russia. In March 2016, a new variant of GII. P16/ GII.4_Sydney_2012 norovirus was first isolated in feces from hospitalized children. In samples from hospitalized adults, this variant was first identified in autumn 2016 (GenBank KY210983, MG892912, and MG892914). GenBank search showed that in the European part of Russia, similar GII.4_ Sydney_2012 noroviruses (GenBank MK033810–MK033811) were found in samples of children from Nizhny Novgorod also at the end of 2016, unfortunately, the RdRp genotype was not determined for those isolates.

Until recently, noroviruses with the GII.P16 RdRp were considered uncommon, although local outbreaks associated with GII.P16/GII.2 (2009/2010 and 2012/2014) were reported in Japan (Iritani et al., 2012; Motomura et al., 2016), and those caused by GII.P16/GII.13 (2009/2010) in Nepal (Hoa-Tran et al., 2015). During the 10-year (2003–2012) monitoring of norovirus genotypes in Novosibirsk, Russia (Zhirakovskaia et al., 2015), GII.P16 RdRp was identified in five samples: GII.P16/GII.16 (GenBank GU292831, KF920739), GII.P16/ GII.3 (GenBank KF944110, KF944111) and GII.P16/GII.5 (GenBank HM596590). Until 2016 in the Russian Federation, except Novosibirsk, GII.P16/GII.3 noroviruses were rarely detected in Omsk (GenBank KT779557, KY362198) and Smolensk (GenBank KF895841), and GII.P16/GII.16 in Moscow and St. Petersburg (GenBank FJ383842, FJ383877). In Novosibirsk, recombinant noroviruses with the novel GII.P16 RdRp, which differed from RdRp of variant 2010–2012 and was in combination with multiple capsid genotypes (GII.13, GII.2, and GII.4_Sydney_2012), were often found in samples from adult AGE patients since 2016 (data not published). Our results confirmed the hypothesis of the spread of newly emerged recombinant norovirus strains with the novel GII. P16 RdRp in different regions of the world (Barreira et al., 2017; Bidalot et al., 2017; Cannon et al., 2017; Choi et al., 2017; Ruis et al., 2017; Hata et al., 2018; Lun et al., 2018).

Before this study, only four complete genome sequences of recombinant GII.P16/GII.3 norovirus strains from Russian were available in the GenBank database (Zhirakovskaia et al., 2015, 2019). In this work, complete genome sequence of the Russian strain Hu/GII.P16-GII.4/RUS/Novosibirsk/ NS16-C38/2016 related to the newly emerged recombinant genotype GII.P16/GII.4_Sydney_2012 was determined. The comparative analysis showed that unique changes occurred in the amino acid sequences of two non-structural proteins – the N-terminal protein p48 and GII.P16 RdRp, as well as in the minor capsid protein VP2; at the same time, no significant changes were detected in the major capsid protein VP1 of GII.4_Sydney_2012.

RNA-dependent RNA polymerase plays a crucial role in the replication of the norovirus genome. Recent studies have shown that the RdRp coding region is changing quickly; however, variable mutation rates were observed in different RdRp genotypes (Ozaki et al., 2018). Our findings are consistent with the hypothesis of Ruiz et al. (2017) that unusual worldwide distribution of the novel GII.P16 lineage is mainly due to changes in the polymerase active center, which could increase the norovirus transmission. However, we assume that changes in the N-terminal protein p48 have also played some role in the wide distribution of these new recombinant strains. It was previously shown that p48 can bind the host restriction factors in an infected cell, allowing norovirus to avoid the host immune response, and its coding region has a higher evolution rate than the complete norovirus genome (Cotten et al., 2014). In addition, it is known that p48 is able to block the local secretory immunity of intestinal epithelial cells, induce the disintegration of the Golgi apparatus and disrupt the intracellular traffic of proteins (Fernandez-Vega et al., 2004; Roth, Karst, 2016). We assume that the insertion of glutamic acid residue into the region, which already contains four consecutive glutamic acid residues, increases the negative charge at the N-terminus of p48, and this may affect both the norovirus entry into the intestinal epithelial cells and the Golgi disintegration rate.

The minor capsid protein VP2, playing an important role in virus replication (Vongpunsawad et al., 2013) and viral particle stability (Lin et al., 2014), is also involved in modulation of the host immune response (Roth, Karst, 2016). The mutation rate of VP2 is higher than that for the major capsid protein VP1 (Cotten et al., 2014). Identified amino acid substitutions could affect the ability of VP2 to suppress the presentation of antigens on cell membranes and the induction of human protective immunity.

## Conclusion

As the result of long-term monitoring of noroviruses RdRp/ VP1 genotypes, the emergence of the novel GII.P16/GII.4_ Sydney_2012 recombinant was recorded in Russia. The analysis showed that the distribution of the newly emerged recombinant GII.P16/GII.4_Sydney_2012 is not associated with changes in the antigenic profile of the major capsid protein VP1, which usually led to the emergence of new epidemic GII.4 variants. In GII.P16/GII.4_Sydney_2012 strains, a certain role was probably played by changes in the minor protein VP2 that might affect the antigenic composition of the viral particle and help to avoid the cellular immune response. In addition, the multiple mutations in two non-structural proteins, the N-terminal protein of p48 and RdRp, probably increased the transmission of noroviruses with the novel GII.P16 RdRp. Further monitoring of genotypes will allow estimation of the spread of emerged recombinant noroviruses with the novel GII.P16 RdRp lineage in the Russian Federation and prediction of their epidemic potential.

## Conflict of interest

The authors declare no conflict of interest.
